# Supplementary material for: Increasing the number of topological nodal lines in semimetals via uniaxial pressure
Source: Sci Rep. 2021 May 19;11:10574. doi: 10.1038/s41598-021-90165-y (PMC8136478; doi:10.1038/s41598-021-90165-y)
Supplement: Supplementary file 1 — Supplementary Information 1. [file 41598_2021_90165_MOESM1_ESM.pdf]

Supplemental material

LATTICE PARAMETER c AS A FUNCTION OF PRESSURE

Figure 1 shows the linear fitting of the lattice parameter c as a function of uniaxial pressure. In black the DFT computed points.

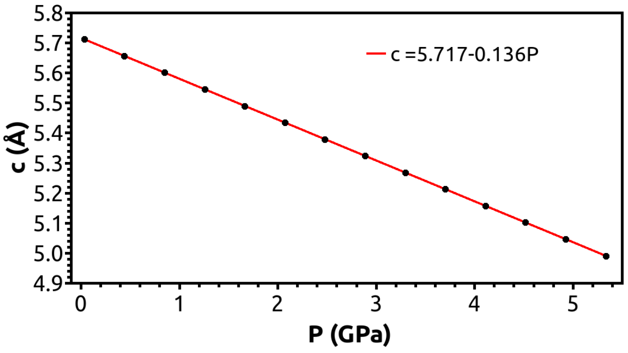

FIG. 1. Linear fitting of lattice parameter c as a function of pressure.

DFT BAND STRUCTURE WITH SOC

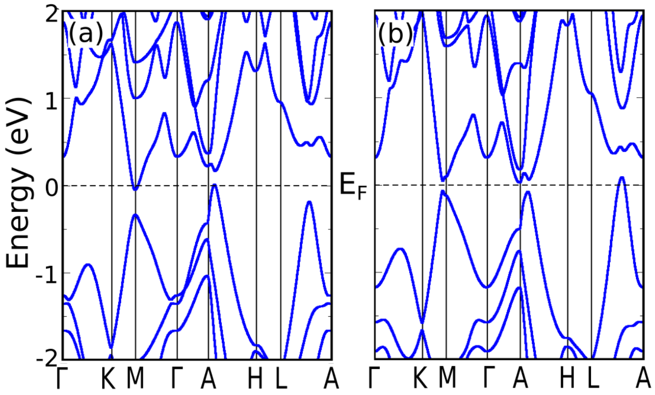

FIG. 2. DFT band structures for $BaSn_2$ including SOC. (a) Band structure for P=0 GPa (T1). (b) Band structure for P=6 GPa (T2)

Figure 2 shows the DFT band structure when SOC is included. SOC was introduced in a second variational manner using the scalar relativistic approximation [1]. At low pressures the band crossing point at the A-H segment (related to the nodal-line) opens and the system becomes a topological insulator (Fig. 2a). For pressures greater than 4 GPa, the crossing around the M point (related to the second nodal-line that appears in the absence of SOC) also opens (Fig. 2b). Therefore, the effect of SOC in both phases is to switch the system into a topological insulator.

TIGHT-BINDING PARAMETERS

The tight-binding (TB) model was built considering that the unit cell of $BaSn_2$ has the following lattice-parameter dependence with uniaxial pressure (P): $a = b = 4.652$ and $c = 5.717 - 0.136P$ Å with P in GPa. The atomic positions in the unit cell are: Ba (0,0,1/2c), Sn1 (2/3a, 1/3a, 0.895c) and Sn2 (1/3a, 1/3a, 0.105c). The Hamiltonian comprises the s and p orbitals of the two Sn atoms in the unit cell and the d orbitals of the Ba atom. Thus, eleven orbitals in total. The on-site energies U of each orbital are given by:

$$\begin{aligned}
U_s &= -5.25 + 0.04P \\
U_{p_{x,y}} &= 0.97 + 0.04P \\
U_{p_z} &= 0.57 + 0.04P \\
U_{d_{z^2}} &= 2.75 + 0.04P \\
U_{d_{xz,yz}} &= 3.63 + 0.04P \\
U_{d_{xy,x^2-y^2}} &= 3.04 + 0.04P,
\end{aligned} \tag{1}$$

in eV and the hopping parameters t between different orbitals are given by:

$$\begin{aligned}
t_{ss} &= 0.4 \\
t_{p_s p_z} &= 0.35 \\
t_{p_s p_{x,y}} &= 1.3 \\
t_{p_z p_z} &= 0.2 \\
t_{p_{x,y} p_{x,y},\sigma} &= 2.0 \\
t_{p_{x,y} p_{x,y},\pi} &= 0.65 \\
t_{p_{x,y} p_z} &= 0.85 \\
t_{d_{z^2} p_z} &= 0.7 \\
t_{d_{xz,yz} p_z} &= 0.7 + 0.2P \\
t_{d_{xy,x^2-y^2} p_z} &= 0.05 \\
t_{d_{z^2} p_{x,y}} &= 0.7 \\
t_{d_{xz,yz} p_{x,y},\sigma} &= 0.3 \\
t_{d_{xz,yz} p_{x,y},\pi} &= 0.22 \\
t_{d_{xy,x^2-y^2} p_{x,y},\sigma} &= 0.24 \\
t_{d_{xy,x^2-y^2} p_{x,y},\pi} &= 0.21
\end{aligned} \tag{2}$$

in eV. Note the σ and π bonding type in some of the hopping parameters. Be aware that these hopping energies have to be multiplied by the Slater-Koster parameters that take into account the relative position between

orbitals [2]. In this TB model that we have built the only t parameters that depend on the pressure are the hopping energies between the Ba d_{xz} and d_{yz} with the Sn p_z orbitals.

SURFACE STATES

The surface states for cuts in different directions are shown in Fig. 3. The effect of the new nodal lines in the high-pressure phase induces a modification of the surface states.

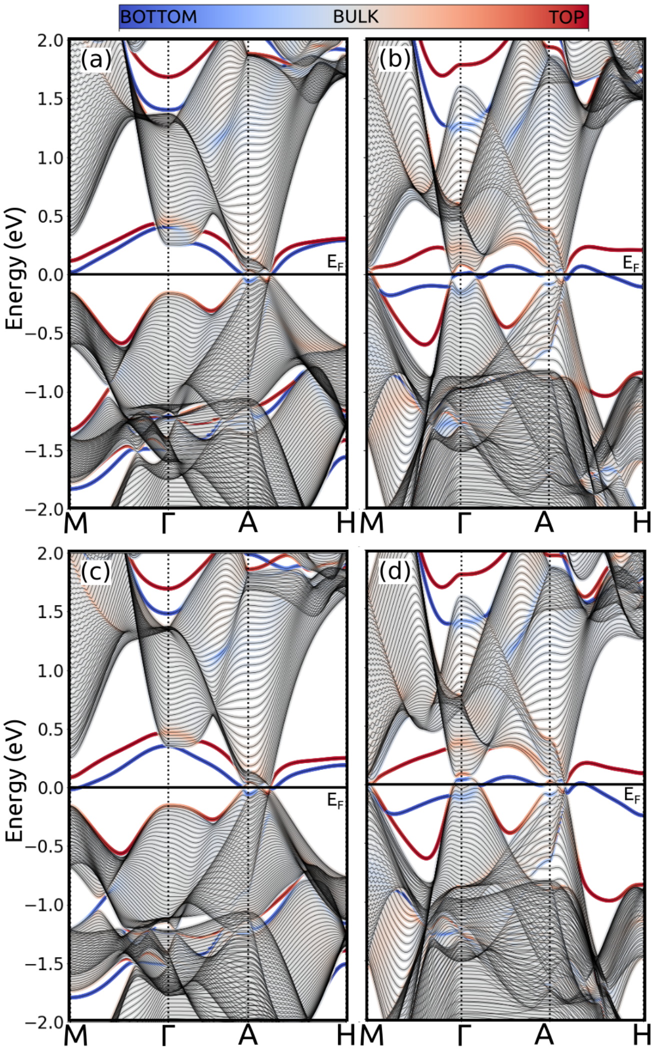

FIG. 3. Energy states in a 50 cell superstructure along the x-direction (a,b) and y-direction (c,d). The colormap represents the expectation value of the position operator along the x-direction and y-direction for (a,b) and (c,d) respectively, in blue (red) the bottom (top) layer and in grey the bulk states. (a,c) For P=0 (T1), and (b,d) for P=6 GPa (T2).

[1] A. H. MacDonald, W. E. Picket, and D. D. Koelling, Journal of Physics C: Solid State Physics **13**, 2675 (1980).

[2] J. C. Slater and G. F. Koster, Phys. Rev. **94**, 1498 (1954).
